# *Mycobacterium haemophilum* Infection after Alemtuzumab Treatment

**DOI:** 10.3201/eid1411.071321

**Published:** 2008-11

**Authors:** Mini Kamboj, Eddie Louie, Timothy Kiehn, Genovefa Papanicolaou, Michael Glickman, Kent Sepkowitz

**Affiliations:** Memorial Sloan-Kettering Cancer Center, New York, New York, USA (M. Kamboj, T. Kiehn, G. Papanicolaou, M. Glickman, K. Sepkowitz); New York University, New York (E. Louie)

**Keywords:** M. haemophilum, infectious complications, alemtuzumab, letter

**To the Editor**: The immunosuppressive agent alemtuzumab is a DNA-derived, humanized monoclonal antibody directed against the panlymphocyte, cell-surface antigen CD52 (*1*). The drug is approved for the treatment of refractory B-cell chronic lymphocytic leukemia (*2*) and also has been used after stem cell (*3*) and organ transplantations (*4*). Alemtuzumab causes profound and prolonged lymphocyte depletion, which results in a variety of complications involving infections (*5*). However, mycobacteria have rarely been reported to cause infection after alemtuzumab treatment. We describe infections with *Mycobacterium haemophilum*, a fastidious nontuberculous mycobacterium, in 2 patients who experienced cutaneous lesions while they received alemtuzumab.

## Patient 1

A 65-year-old man with refractory chronic lymphocytic leukemia had been receiving treatment with alemtuzumab for 3 months. During a 5-week period beginning 15 weeks after the alemtuzumab therapy started, 20–30 tender nodular-ulcerative lesions developed on the patient’s extremities. Most of the lesions were distributed along a saphenous vein site (Figure). Immediately before receiving alemtuzumab, he had been given rituximab for 3 months. A punch biopsy of the cutaneous lesion showed lymphogranulomatous inflammation in the dermis. Acid-fast stains of the skin punch biopsy specimen, as well as aspirated material from the lesions, demonstrated acid-fast bacilli. Cultures on Middlebrook 7H11 agar (Becton Dickinson and Company, Sparks, MD, USA) containing X-factor strips incubated at 30°C showed growth of the acid-fast bacilli after 13 days. The isolate was subsequently identified as *M. haemophilum* by using conventional biochemical profiles and assessment of morphologic features, including an optimal growth temperature of 30°C and a hemin requirement. The patient was treated with 4 drugs (rifampin, doxycycline, clarithromycin, ciprofloxacin), and he rapidly improved. Susceptibility testing, using broth MIC determinations described in Clinical and Laboratory Standards Institute publication M-24A (www.clsi.org/source/orders/free/m24-aa.pdf), indicated that the isolate was sensitive to clarithromycin, ciprofloxacin, clofazimine, and linezolid; intermediately sensitive to rifampin; but resistant to rifabutin, doxycycline, ethambutol, streptomycin, and amikacin. The antimicrobial drugs the patient was receiving were changed to only rifampin, clarithromycin, and ciprofloxacin. He completed a 6-month course of treatment course without recurrence of the lesions.

**Figure:**
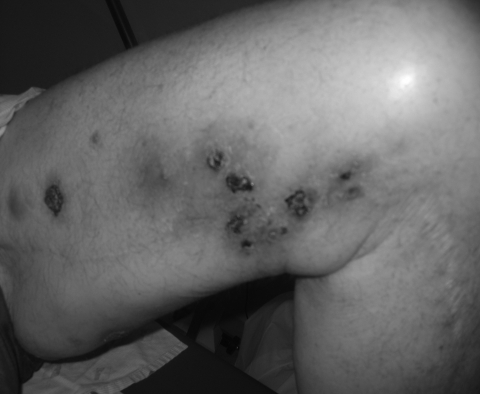
Nodular-ulcerative skin lesions on the left thigh caused by *Mycobacterium haemophilum* infection in a patient with chronic lymphocytic leukemia (patient 1) whose condition had been treated with alemtuzumab.

## Patient 2

A 17-year-old woman with severe systemic lupus erythematosus and secondary myelodysplastic syndrome received an unrelated T-cell depleted bone marrow transplant. Her conditioning regimen included melphalan, thiotepa, fludarabine, and 2 doses of alemtuzumab. She initially did well posttransplant and was discharged from the hospital Approximately 3 months later, 40–50 tender erythematous papular lesions developed on her extremities. A skin biopsy specimen showed mycobacterial panniculitis. Cultures from skin, blood, and bone marrow grew *M. haemophilum* after 18–19 days’ incubation. She was successfully treated with rifampin, clarithromycin, and gatifloxacin; however, she died several months later from unrelated complications.

*M. haemophilum* was first described in 1978 when it was isolated from cutaneous lesions of a woman from Israel with Hodgkin disease (*6*). *M. haemophilum* most often causes joint, cutaneous, and pulmonary infections in immunocompromised patients (*7*) and lymphadenitis in immunocompetent children (*8*). *M. haemophilum* is a fastidious organism that requires media supplemented with ferric ions in the form of hemin, hemoglobin, or ferric ammonium citrate, and incubation at 30°C–32°C for several weeks. On the basis of our experience at Memorial Sloan-Kettering Cancer Center (23 cases of *M. haemophilum* infection observed from 1990 through 2000) (*9*), the following specimens are routinely set up for culture: blood smear specimens that are positive for acid-fast bacilli, synovial or joint fluids, skin biopsy specimens, cutaneous lesions, ulcers, abscesses, lymph nodes, and lung biopsy specimens. Culture media include Middlebrook 7H11 agar plates with a hemin-containing paper strip (X-factor) placed on the agar surface that are then incubated at 30°C for 6 weeks. Growth of the organism is usually detected within 2 to 3 weeks, and the isolates are usually susceptible in vitro to the quinolones, macrolides, and rifamycins and resistant to several drugs for tuberculosis, including ethambutol, isoniazid, and pyrazinamide (*9*).

Alemtuzumab has been associated with the development of infections caused by a variety of microorganisms. However, mycobacteria have infrequently been the reported cause. In a review of 547 organ transplant recipients who received alemtuzumab treatment, miliary tuberculosis developed in 1 recipient of a kidney transplant, and pulmonary infection with *M. kansasii* developed in 2 recipients of lung transplants (*5*). There is also a case report of systemic *M. bovis* infection developing in a patient with relapsing B chronic lymphocytic leukemia after administration of alemtuzumab (*10*).

Although we believe that alemtuzumab is responsible for the severe immunosuppression that predisposed these patients to *M. haemophilum* infection, other explanations are plausible. For example, patient 1 had received rituximab and cyclophosamide for 6 months. These drugs, in addition to his underlying disease of chromic lymphocytic leukemia, may have predisposed him to *M. haemophilum* infection. However, his lesions did not appear until he received alemtuzumab. In patient 2, the immunosuppression associated with his transplant may have predisposed the patient to *M. haemophilum* infection.

This report identifies *M. haemophilum* as an opportunistic pathogen in patients who have received alemtuzumab. We recommend that all patients who have received at least 1 dose of alemtuzumab, and who have undiagnosed tender skin lesions located over the extremities, be evaluated by using appropriate techniques to isolate *M. haemophilum.* Communication with microbiology laboratory staff concerning appropriate methods for detection of the organism is crucial.
